# Pathology-guided multimodal transformer predicts postoperative survival benefit from adjuvant chemotherapy in glioma: from DNA damage repair to immune characterization

**DOI:** 10.3389/fimmu.2026.1845251

**Published:** 2026-10-16

**Authors:** Zehui Li, Xin Duan, Junjie Lian, Xuan Li, Qian Liang, Xu Liu, Yan Tan, Guoqiang Yang, Jiangfeng Du, Lina Wu, Xiaochun Wang, Hui Zhang

**Affiliations:** 1 Department of Radiology, First Hospital of Shanxi Medical University, Taiyuan, Shanxi, China; 2 College of Medical Imaging, Shanxi Medical University, Taiyuan, Shanxi, China; 3 Intelligent Imaging Big Data and Functional Nano-imaging Engineering Research Center of Shanxi Province, First Hospital of Shanxi Medical University, Taiyuan, Shanxi, China; 4 Department of Pathology, First Hospital of Shanxi Medical University, Taiyuan, Shanxi, China

**Keywords:** digital pathology, high-grade glioma, magnetic resonance imaging, survival benefit, temozolomide, transformer

## Abstract

**Introduction:**

High-grade gliomas (HGGs) are aggressive primary brain tumors with high recurrence rates and poor prognosis. Survival benefit from adjuvant temozolomide (TMZ) varies substantially across patients, and reliable preoperative biomarkers for treatment stratification remain limited. This study aims to develop an interpretable multimodal deep learning model integrating whole-slide histopathological images and multiparametric MRI to preoperatively predict survival benefit from adjuvant TMZ in HGG patients.

**Methods:**

A Transformer-based multimodal fusion model was proposed. Key tumor regions were identified from whole-slide pathology images via weakly supervised clustering, and pathological features were extracted using attention-based multiple instance learning. Concurrently, 2.5D tumor subregional features were derived from multiparametric MRI sequences. These multimodal features were deeply fused using a Transformer encoder. The model was developed and validated on a multicenter cohort (504 patients), and its biological basis was explored through transcriptomic analysis, gene set enrichment analysis, protein-protein interaction networks, and immune deconvolution.

**Results:**

The fusion model achieved AUCs of 0.910, 0.861, and 0.833 in the training, internal validation, and external validation cohorts, respectively. Transcriptomic analysis implicated DNA damage repair processes in high-benefit patients, suggesting that enhanced DNA repair capacity may contribute to TMZ sensitivity. Immune deconvolution showed higher M1 macrophage infiltration and lower regulatory T cell abundance in the high-benefit group, consistent with a less immunosuppressive tumor microenvironment. Importantly, low-benefit patients who completed TMZ chemotherapy had shorter survival than those treated with concurrent chemoradiotherapy alone or surgery alone.

**Conclusion:**

This model identifies patients unlikely to benefit from standard TMZ chemotherapy, providing a tool for postoperative personalized treatment stratification and optimization of therapeutic strategies in HGGs.

## 1 Introduction

High-grade gliomas (HGGs) are the most common primary malignant tumors of the central nervous system, characterized by aggressive invasiveness and high recurrence rates (1, 2). Currently, postoperative adjuvant chemotherapy with temozolomide (TMZ) represents the standard treatment regimen; however, significant inter-individual variability in survival benefit has been observed among patients (3, 4). Therefore, accurately identifying patients with low survival benefit from standard adjuvant TMZ chemotherapy is of great importance for optimizing postoperative treatment strategies and promoting more precise therapeutic decision-making.

Recent advances in digital pathology and medical imaging have created new opportunities for tumor prognosis prediction (5–7). Whole-slide images (WSIs) provide detailed microstructural tissue information about tumors, while magnetic resonance imaging (MRI) captures their macroscopic morphological and functional characteristics. However, effectively integrating these multimodal data and extracting biologically meaningful features remains a challenge. Traditional pathological feature analysis predominantly relies on manual assessment, which is characterized by limitations such as high subjectivity and poor reproducibility (8, 9). Although deep learning techniques have demonstrated substantial capability in automated feature extraction (10), the high resolution and pronounced heterogeneity of WSIs render direct analysis of entire slides highly challenging. Multiple instance learning (MIL) partially addresses this issue by treating WSIs as collections of patches (11, 12); however, identifying discriminative regions from vast numbers of patches remains a challenge. Furthermore, the success of Transformer models in natural language processing and computer vision has demonstrated their ability to capture long-range dependencies and complex patterns, offering new insights for integrating multimodal medical data (13–15). Nevertheless, existing studies have largely focused on single modalities or simple feature concatenation, failing to fully exploit the intrinsic correlations among pathological data, imaging data, and biological information.

Therefore, this study proposes a multimodal fusion model based on a Transformer architecture to predict postoperative survival benefit from adjuvant TMZ chemotherapy in patients with HGGs, providing a reference for subsequent personalized treatment decision-making at the postoperative, pre-adjuvant-treatment clinical decision point, where both surgical pathology specimens and preoperative MRI data are already available. First, we employed weakly supervised clustering to select patches from WSIs, identifying key pathological regions associated with malignant tumor progression, including the tumor-infiltrating margin, microvascular proliferation zone, and necrotic area. These regions also serve as critical interfaces for tumor-immune interactions within the glioma immune microenvironment. Pathological features were then aggregated using an attention mechanism. Subsequently, we extracted MRI features and achieved deep fusion of pathological and imaging features through a Transformer encoder. Finally, survival benefit was predicted using the fused multimodal features.

## 2 Materials and methods

### 2.1 Patients

This study enrolled 504 HGG patients with complete preoperative MRI (CE-T1WI, T2-FLAIR), postoperative pathology, and clinical data. Of these, 422 patients were from three centers (First Hospital of Shanxi Medical University, Shanxi Provincial People’s Hospital, and Bethune Hospital of Shanxi Province; 2011–2023) and were pathologically confirmed as WHO grade 3–4 gliomas. These patients were divided into an adjuvant chemotherapy group, a concurrent chemoradiotherapy alone group, and a surgery alone group. The adjuvant chemotherapy group was split 7:3 into training and internal validation sets, while the other two groups were used for survival validation. Additionally, 82 patients from The Cancer Imaging Archive (TCIA) were included for external validation after excluding 324 of the 406 initial cases. Low chemotherapy benefit was defined as overall survival of less than 14 months, and high benefit as greater than 14 months. The 14-month overall survival threshold was selected based on the landmark EORTC-NCIC trial, which reported a median overall survival of approximately 14–15 months for patients receiving standard concurrent radiotherapy and temozolomide regimen (16). Detailed inclusion and exclusion criteria, MRI acquisition and pathology slide processing protocols, and molecular testing methodology (MGMT and IDH) are provided in the **Supplementary Materials**.

### 2.2 WSI preprocessing and patch feature extraction

We implemented a systematic preprocessing strategy by segmenting WSIs into 512×512-pixel patches at 20× magnification to prioritize high-quality data acquisition. A recognition module based on a convolutional neural network (CNN) was employed to exclude invalid regions, including pure background, blurry areas, artifacts, and tissue-deficient regions, ultimately yielding 3,694,856 patches. Color normalization was first performed using the Vahadane algorithm, followed by Z-score standardization across RGB channels to achieve consistent intensity values. For patch-level feature extraction, we employed CNN architectures, specifically ResNet-18, ResNet-34, and ResNet-50, to extract deep features, with each patch represented by a 2048-dimensional feature vector. To improve model performance, a transfer learning approach was adopted by initializing the networks with pre-trained weights from ImageNet, enabling effective reuse of learned general features. During the training phase, optimization was performed using stochastic gradient descent (SGD) with a cosine annealing scheduler to dynamically adjust the learning rate, starting at 0.01.

### 2.3 Weakly supervised clustering and pathological validation

To identify biologically meaningful tissue patterns in WSIs, we applied K-means clustering to deep features extracted from all patches. A majority voting strategy was used to aggregate subregion predictions and generate WSI-level classifications, thereby enabling the selection of optimal clustering models. This approach extracted morphological features via deep learning, facilitating the comparison of patch-level similarities and differences while revealing their spatial distribution characteristics and clinical relevance. Based on independent evaluation by neuropathologists considering typical glioma pathological features, including tumor cell density, necrotic areas, and microvascular proliferation, patches were ultimately clustered into five pathologically meaningful categories. This expert validation ensured alignment with established clinical diagnostic criteria.

### 2.4 Pathological clustering region selection strategy

We first trained three CNN classifiers (ResNet-18, ResNet-34, and ResNet-50) for each of the five patch clusters across all slides in the training set, using a batch size of 32 over 50 epochs. In the validation set, we optimized the classifiers and employed a voting strategy to estimate each cluster’s classification accuracy. Additionally, we incorporated neuropathologists’ opinions regarding histopathological distribution characteristics and the performance of each cluster across different models. Based on this comprehensive evaluation, we selected the most relevant clusters for further analysis. To assess the effectiveness of this strategy, patches selected by our method were integrated into the electronic annotation workflow. Weakly supervised learning was then performed on the annotated regions using ResNet-50, with the same processing applied to all patches. The effectiveness of the pathological clustering region selection strategy was validated by comparing accuracy and the area under the curve (AUC) of the receiver operating characteristic (ROC) curve.

### 2.5 Attention-based multiple instance learning feature aggregation

To address the challenge posed by the large number of patches and their complex spatial distribution in WSIs, we designed an attention-based MIL framework that aggregates thousands of patch features from each patient into a unified patient-level representation. In this framework, each WSI is treated as a “bag” containing patches as “instances”. The attention mechanism automatically learns the importance weight of each patch with respect to the final prediction, which is mathematically expressed as follows:

For patient  i with $N_{i}$ patches, let $\;{\text{h}}_{j}\in {\mathbb{R}}^{2048}\;$denote the feature representation of the j-th patch. The attention weight ${\text{a}}_{\text{j}}$ is computed through a two-layer neural network:


$$a_{j}=softmax({\text{w}}^{⊤}\tanh(\text{V}{\text{h}}_{j}^{⊤}))$$

where V and w are learnable parameters. The overall patient feature representation is obtained by weighted summation:


$${\text{z}}_{i}=\sum_{j=1}^{N_{i}}a_{j}{\text{h}}_{j}$$

Finally, the feature dimension is reduced to 512 through a linear transformation, yielding a compact patient-level feature representation ${\text{ x}}_{\text{i}}\in {\mathbb{R}}^{512}$.

### 2.6 MRI preprocessing and feature extraction

Regions of interest (ROIs) on MRI images were manually delineated using ITK-SNAP (version 3.6.0). Tumor regions were first identified on CE-T1WI images and subsequently registered with T2-FLAIR images for further analysis. ROIs were outlined by neuroradiologists with over 10 years of experience. When necessary, a senior neuroradiologist with more than 20 years of experience provided additional guidance to ensure accuracy and consistency. The axial slice containing the maximum tumor area from each sequence was selected as the “maximum tumor image.” Four additional slices were extracted: two slices above (+2), one slice above (+1), two slices below (–2), and one slice below (–1) relative to the maximum tumor image. These slices were then fused with label information to construct a 2.5D multi-channel input. A ResNet-50 convolutional neural network pre-trained on the ImageNet dataset served as the feature extractor, generating 2048-dimensional deep feature representations for each slice. Principal component analysis (PCA) was subsequently applied to compress these 2048-dimensional features into 512 principal components.

### 2.7 Transformer fusion model

In recent years, the Transformer model has garnered significant attention in medical binary classification tasks (17–19). Although originally developed for natural language processing, its self-attention mechanism has demonstrated remarkable advantages in processing complex structured data. The Transformer model employed in this study integrates structured inputs from both pathology and imaging modalities, with each sample represented as a 512-dimensional feature vector and organized in bags containing two samples. The model architecture comprises two layers of Transformer encoders, each equipped with eight attention heads, an embedding dimension of 1024, and multilayer perceptron layers further expanded to 2048 dimensions. The AdamW optimizer was selected with a fixed initial learning rate of 0.01. This architecture leverages the nonlinear mapping capabilities and feature interaction mechanisms of Transformers to perform deep transformation and enhancement of patient-level features aggregated by MIL. Residual connections and layer normalization ensure training stability, with final classification predictions generated through global pooling and fully connected layers. **Figure 1** illustrates the complete workflow of the fusion model.

**Figure 1:**
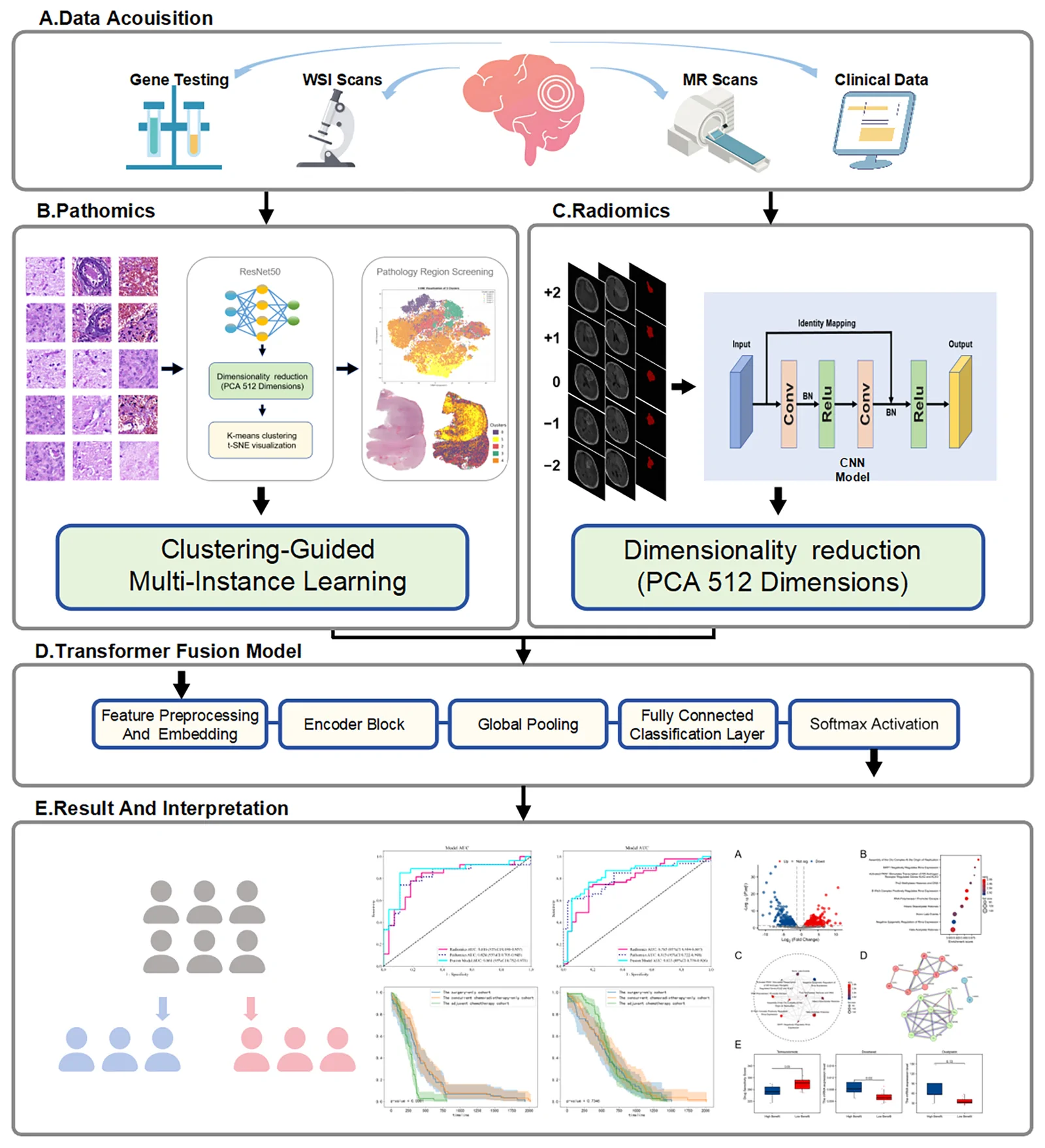
Overall workflow and pipeline of the study. **(A)** Data acquisition from gene testing, WSI scanning, MRI scanning, and clinical data. **(B)** Pathomics analysis, including image patching, feature extraction, dimensionality reduction, K-means clustering, and pathological region selection. **(C)** Radiomics analysis, including 2.5D multi-slice construction from CE-T1WI and T2-FLAIR, CNN-based feature extraction, and dimensionality reduction. **(D)** Transformer fusion model, including preprocessing, encoding, pooling, classification, and softmax activation. **(E)** Results and interpretation. Patients were stratified into high- and low-benefit groups; model discrimination, survival differences, and biological mechanisms were evaluated accordingly.

### 2.8 Survival validation

To address the ethical challenge of prospectively validating chemotherapy benefit in glioma patients, we designed a survival validation framework based on the Transformer fusion model. Three patient groups were enrolled: surgery alone, postoperative chemoradiotherapy alone, and complete adjuvant temozolomide chemotherapy. Preoperative MRI and postoperative pathological slides were used to predict high or low chemotherapy benefit. Using Kaplan-Meier analysis, we compared the survival of low-benefit patients identified by the model with that of patients in the two control groups who did not receive full adjuvant TMZ but were also classified as low-benefit. This validated the model’s ability to identify low-benefit patients and supported decision-making for postoperative treatment.

#### 2.9 Biological validation

To investigate the molecular mechanisms underlying the deep learning model’s predicted TMZ response, we performed bioinformatics analysis on a transcriptomic subset (n = 43) derived from the 82-patient external validation cohort (detailed clinical characteristics reported in **Supplementary Table S8**), comprising patients with available RNA-seq data from TCGA-GBM (n = 27) and TCGA-LGG (n = 16). The specific composition of this transcriptomic subset is provided in **Supplementary Table S9**. All patients received standard treatment according to their respective TCGA protocols. Based on model predictions, patients were divided into high-benefit (n = 32) and low-benefit (n = 11) groups. Bioinformatics analysis was conducted using R software (version 4.2.1). Gene set enrichment analysis (GSEA) was performed on gene expression data using the clusterProfiler package (version 4.4.4). Gene sets were obtained from the MSigDB database (specifically the c2.cp.all.v2022.1.Hs.symbols.gmt collection, comprising 3,050 canonical pathways). Pathways with both adjusted p-values (p.adjust) < 0.05 and false discovery rates (qvalue) < 0.25 were considered significantly enriched. To further identify key regulatory genes, we extracted core enrichment genes from all significantly enriched pathways and calculated the occurrence frequency of each gene across core gene sets, focusing on non-histone genes with an occurrence frequency exceeding 20. To explore interactions and functional modules among these genes, the selected high-frequency non-histone genes were submitted to the STRING database to construct a protein-protein interaction (PPI) network (confidence > 0.9), analyze network topological properties, and identify functional modules.

#### 2.10 Statistical analysis

All statistical inferences and model development were performed using Python 3.9.0. Two-tailed hypothesis tests with P < 0.05 were considered statistically significant. Continuous variables were summarized as mean ± standard deviation, and inter-group differences were assessed using the non-parametric Kruskal-Wallis H test. Categorical variables were presented as frequencies (percentages) and analyzed using Pearson’s chi-square test. Model discriminative ability was evaluated using ROC curves, with performance metrics including AUC, accuracy, sensitivity, and specificity. The DeLong test was used to compare AUCs across models. Survival outcomes were estimated using the Kaplan-Meier method, and group differences were assessed using the log-rank test.

## 3 Results

### 3.1 Patients

This study enrolled five cohorts: training set (n = 143), internal validation set (n = 54), external validation set (n = 82), surgery alone group (n = 107), and concurrent chemoradiotherapy group (n = 118). **Table 1** summarizes the baseline characteristics across cohorts. Regarding baseline characteristics, no statistically significant differences were observed across cohorts in sex, tumor margin, cystic necrosis, midline shift, or overall survival (OS ≤ 14 months: 42.68%–55.24%) (all P > 0.05). Sex distribution was highly consistent (female: 55.56%–61.68%), tumor margins were predominantly “unclear” (76.83%–85.59%), most cohorts were dominated by “absence of midline shift” (71.95%–94.44%), and OS distribution was similar, indicating good balance in these baseline features. Mean age ranged from 49.15 to 55.65 years, with no significant inter-group difference (P = 0.074). Only WHO grade showed a statistically significant difference among the five cohorts (P = 0.049), with the external validation set having the highest proportion of grade IV tumors (81.71%), followed by the training set (74.83%), possibly attributable to the external validation set originating from the TCIA database. Among all 504 patients across five cohorts, MGMT methylation and IDH mutation data were available for 422 patients from the local centers, with no significant differences in distribution across cohorts (MGMT: P = 0.405; IDH: P = 0.964; **Supplementary Table S3**). In the adjuvant chemotherapy cohort (n = 197), log-rank tests confirmed significant survival differences by MGMT (P = 0.003) and IDH status (P < 0.001; **Supplementary Figure S2**).

**Table 1:** Basic clinical characteristics of patients in the 5 cohorts.

Patient characteristic	Training cohort (n = 143)	Internal validation cohort(n=54)	External validation cohort (n=82)	The surgery-only cohort (n=107)	The concurrent chemoradiotherapy-only cohort (n=118)	*P* value
Age (Mean ± SD)	51.58 ± 13.48	51.44 ± 14.20	55.65 ± 13.49	52.35 ± 16.47	49.15 ± 14.58	0.074
Gender						0.998
Male	63(44.06%)	24(44.44%)	36(43.90%)	41(38.32%)	48(40.68%)	
Female	80(55.94%)	30(55.56%)	46(56.10%)	66(61.68%)	70(59.32%)	
WHO grading						0.049
WHO III	36(25.17%)	20(37.04%)	15(18.29%)	42(39.25%)	42(35.59%)	
WHO IV	107(74.83%)	34(62.96%)	67(81.71%)	65(60.75%)	76(64.41%)	
Tumor margins						0.813
Clear	28(19.58%)	11(20.37%)	19(23.17%)	19(17.76%)	17(14.41%)	
Unclear	115(80.42%)	43(79.63%)	63(76.83%)	88(82.24%)	101(85.59%)	
Cystic necrosis						0.867
none	7(4.90%)	2(3.70%)	6(7.32%)	5(4.67%)	7(5.93%)	
mild	14(9.79%)	6(11.11%)	11(13.41%)	9(8.41%)	13(11.02%)	
moderate	22(15.38%)	10(18.52%)	10(12.20%)	21(19.63%)	16(13.56%)	
severe	100(69.93%)	36(66.67%)	55(67.07%)	72(67.29%)	82(69.49%)	
Midline shift						0.120
Yes	23(16.08%)	3(5.56%)	23(28.05%)	13(12.15%)	16(13.56%)	
No	120(83.92%)	51(94.44%)	59(71.95%)	94 (87.85%)	102(86.44%)	
OS						0.192
≤ 14 mo	79(55.24%)	27(50.00%)	35(42.68%)	55(51.40%)	60(50.85%)	
> 14 mo	64(44.76%)	27(50.00%)	47(57.32%)	52(48.60%)	58(49.15%)	

### 3.2 Histology-based tumor classification

After image cropping and color normalization preprocessing, a total of 3,694,856 independent image patches were obtained. To evaluate the impact of lesion-region delineation on deep learning model performance, we implemented a multi-step analytical pipeline. First, deep learning features were extracted from all patches using a ResNet-50 model, and the resulting features were reduced to 512 dimensions via principal component analysis. Second, K-means clustering was applied to partition the patches into five categories, with visualization performed using t-SNE (**Figures 2A, B**). To validate the histological specificity of the clustering results, we conducted visual validation at the whole-slide image level (**Figure 2C**). Analysis of H&E-stained sections revealed that cluster 0 corresponded to the tumor-infiltrating margin and reactive gliosis region, cluster 1 represented the high cellular density tumor area, cluster 2 corresponded to the microvascular proliferation zone, cluster 3 represented the mixed region of low cellular density tumor and normal tissue, and cluster 4 corresponded to the tumor necrosis area (**Figure 2D**). Subsequently, weakly supervised deep learning was performed using CNN architectures, including ResNet-18, ResNet-34, and ResNet-50. It was observed that although larger networks effectively identified clusters 1 and 3 (high cellular density tumor area and mixed low cellular density tumor/normal tissue region), their risk-stratification accuracy was paradoxically reduced (**Figure 3A**). Therefore, we developed a pipeline for pathological region selection that excluded clusters 1 and 3, focusing instead on more representative regions within the tumor microenvironment.

**Figure 2:**
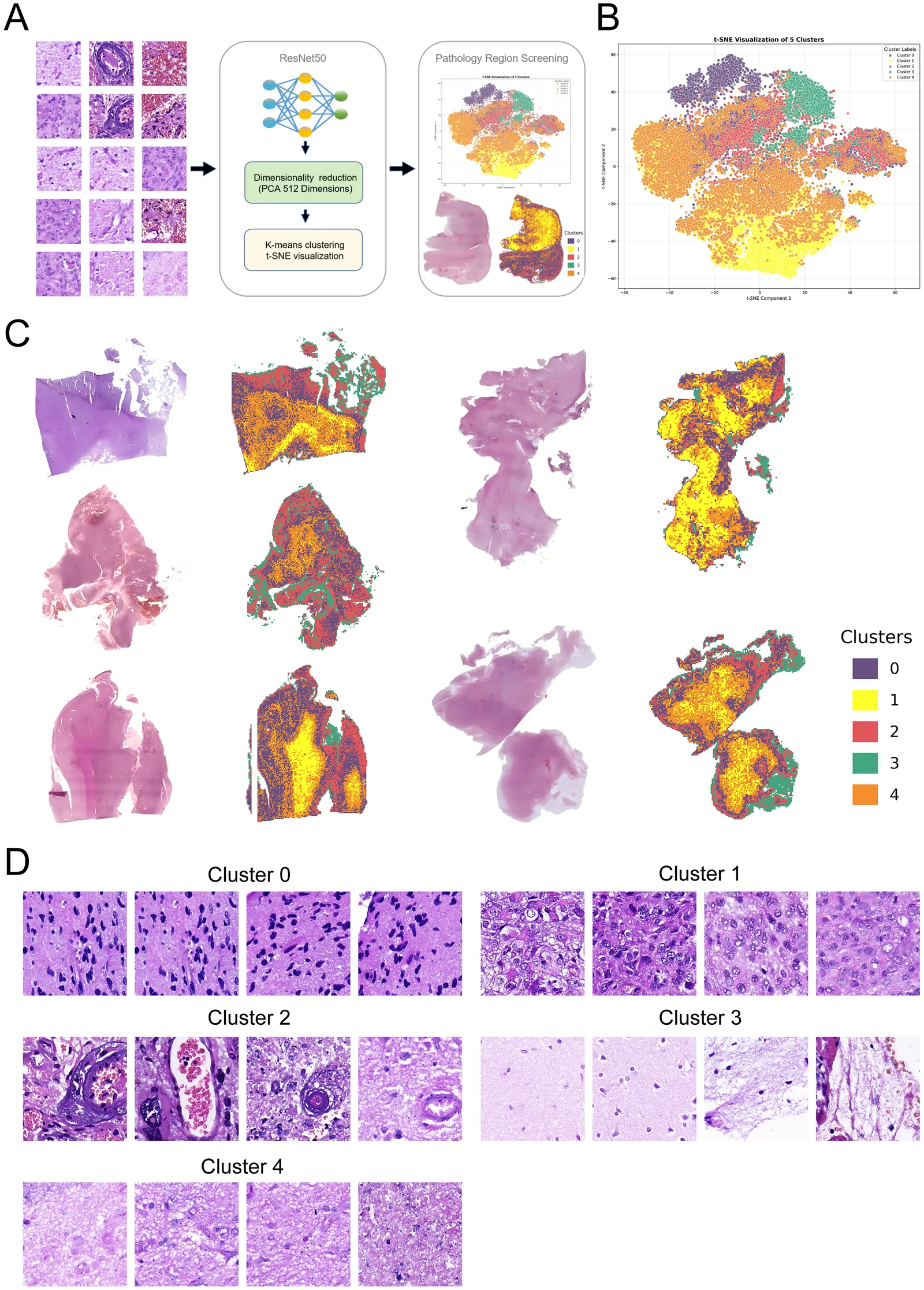
Clustering and spatial visualization of pathological regions in H&E-stained whole-slide images. **(A)** Workflow for pathological region analysis: feature extraction from image patches using ResNet50, followed by PCA dimensionality reduction, K-means clustering, and pathological annotation. **(B)** t-SNE two-dimensional visualization of K-means clustering results based on image patch features. **(C)** Spatial distribution of clustered patches within the WSI. **(D)** Representative image patches for each cluster.

**Figure 3:**
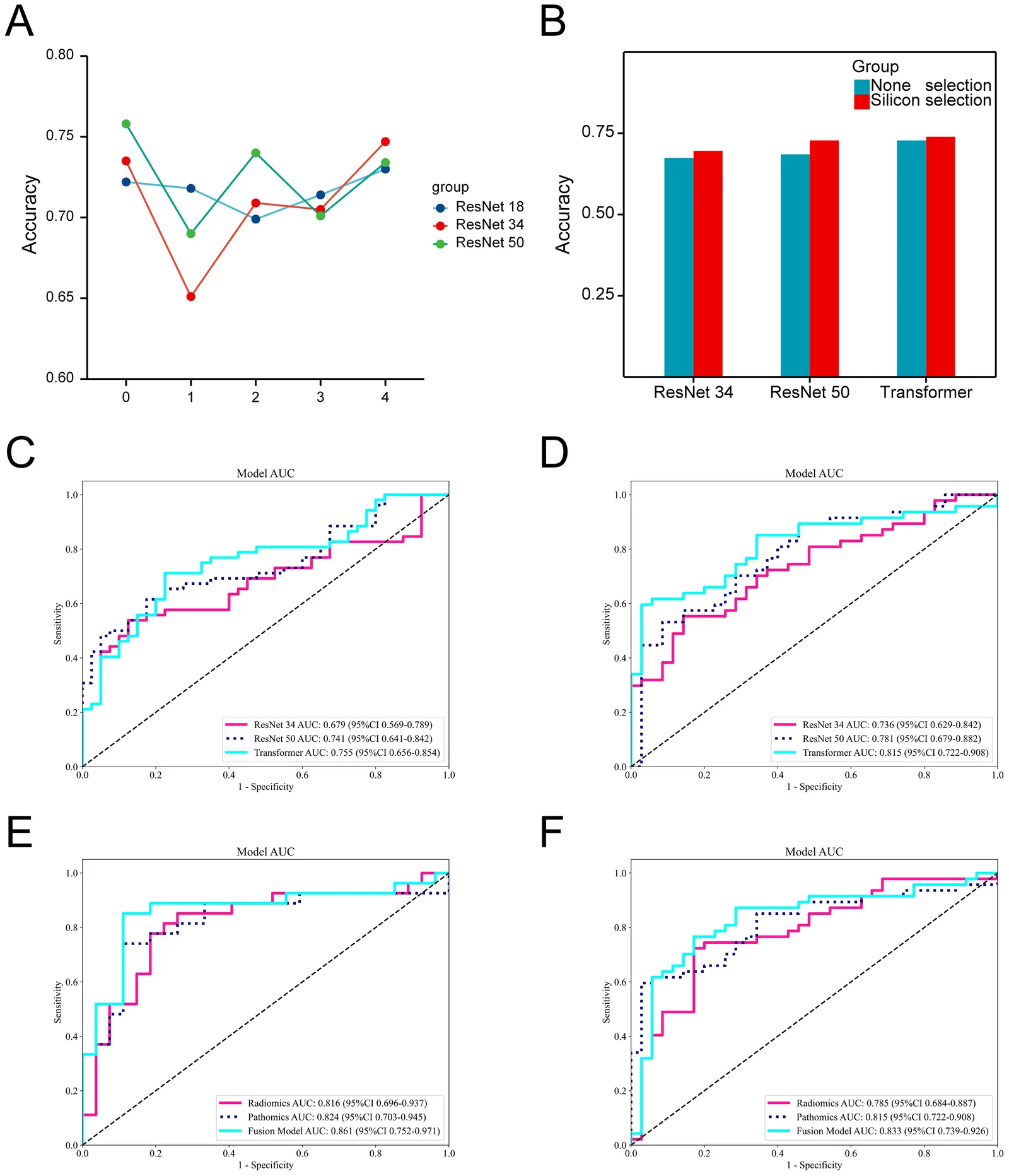
Performance comparison of weakly supervised deep learning models with and without lesion region selection. **(A)** Line plot of prediction accuracy for each lesion cluster using different CNN models (ResNet18, ResNet34, ResNet50) and a voting method. **(B)** Bar chart comparing the accuracy of different deep learning algorithms under the two conditions. **(C, D)** ROC curves and AUC values for multiple deep learning models, corresponding to scenarios with **(C)** and without **(D)** lesion region selection. **(E, F)** ROC curves and AUC values for Radiomics, Pathomics, and Fusion models on the test and validation sets under the lesion region selection condition.

### 3.3 Model development and performance

For each patient, 2,048 deep learning features were extracted from each pathological patch and 2.5D multi-slice MRI. Before Transformer-based fusion, these feature sets were reduced to 512 dimensions using MIL and PCA. Models incorporating the pathological region selection strategy achieved significantly higher accuracy across various deep learning architectures (**Figure 3B**). Transformer models based on radiomic and pathomic features also demonstrated improved AUC and predictive performance (**Figures 3C, D**). **Table 2** summarizes performance metrics for all cohorts. The integrated Transformer model achieved superior AUCs: 0.910 in the training cohort, 0.861 in the internal validation cohort, and 0.833 in the external validation cohort (**Figures 3E, F**). Decision curve analysis revealed a higher net benefit for the integrated model, confirming its clinical relevance and potential for enhanced predictive efficacy. Comprehensive DCA and calibration curves for all cohorts are presented in **Supplementary Figure S1**.

**Table 2:** Performances of the predictive models in the study sets.

Model	Cohort	ACC	AUC(95%CI)	SEN	SPE
Radiomics	Train	0.888	0.951(0.919 - 0.983)	0.797	0.962
Pathomics	Train	0.853	0.902(0.852 - 0.951)	0.750	0.937
Fusion model	Train	0.860	0.910(0.862 - 0.958)	0.719	0.975
Radiomics	test	0.796	0.816(0.696 - 0.937)	0.778	0.815
Pathomics	test	0.815	0.824(0.704 - 0.945)	0.741	0.889
Fusion model	test	0.870	0.861(0.752 - 0.971)	0.852	0.889
Radiomics	val	0.768	0.785(0.684 - 0.886)	0.723	0.829
Pathomics	val	0.756	0.815(0.722 - 0.908)	0.696	0.871
Fusion model	val	0.793	0.833(0.739 - 0.926)	0.766	0.829

AUC, area under the ROC curve; ACC, accuracy; SEN, sensitivity; SPE, specificity.

Analysis of correctly predicted low-benefit patients in the test set (**Figure 4A**) revealed lesions exhibiting tumor cell atypia, altered microvascular morphology, and residual necrotic areas. Pathological Grad-CAM heatmaps showed activation concentrated at the infiltrative tumor margin—a region often enriched in immunosuppressive cells, such as tumor-associated macrophages and regulatory T cells, in HGGs—with more pronounced cellular atypia and a denser tumor cell distribution. Imaging Grad-CAM heatmaps demonstrated significantly enhanced and broader activation in regions corresponding to low-benefit predictions (**Figures 4B, C**), suggesting higher biological aggressiveness associated with poor clinical outcomes. Conversely, analysis of correctly predicted high-benefit patients (**Figure 5A**) revealed morphological features indicating lower atypia, regular microvascular morphology, and sparse residual cells in necrotic areas. Imaging Grad-CAM heatmaps showed activation predominantly concentrated in peritumoral edema regions (**Figures 5B, C**). In HGGs, functional immune cell infiltration is relatively preserved in this region, suggesting relatively localized tumor growth and a microenvironment favorable for antitumor immune responses, both of which are associated with better clinical outcomes.

**Figure 4:**
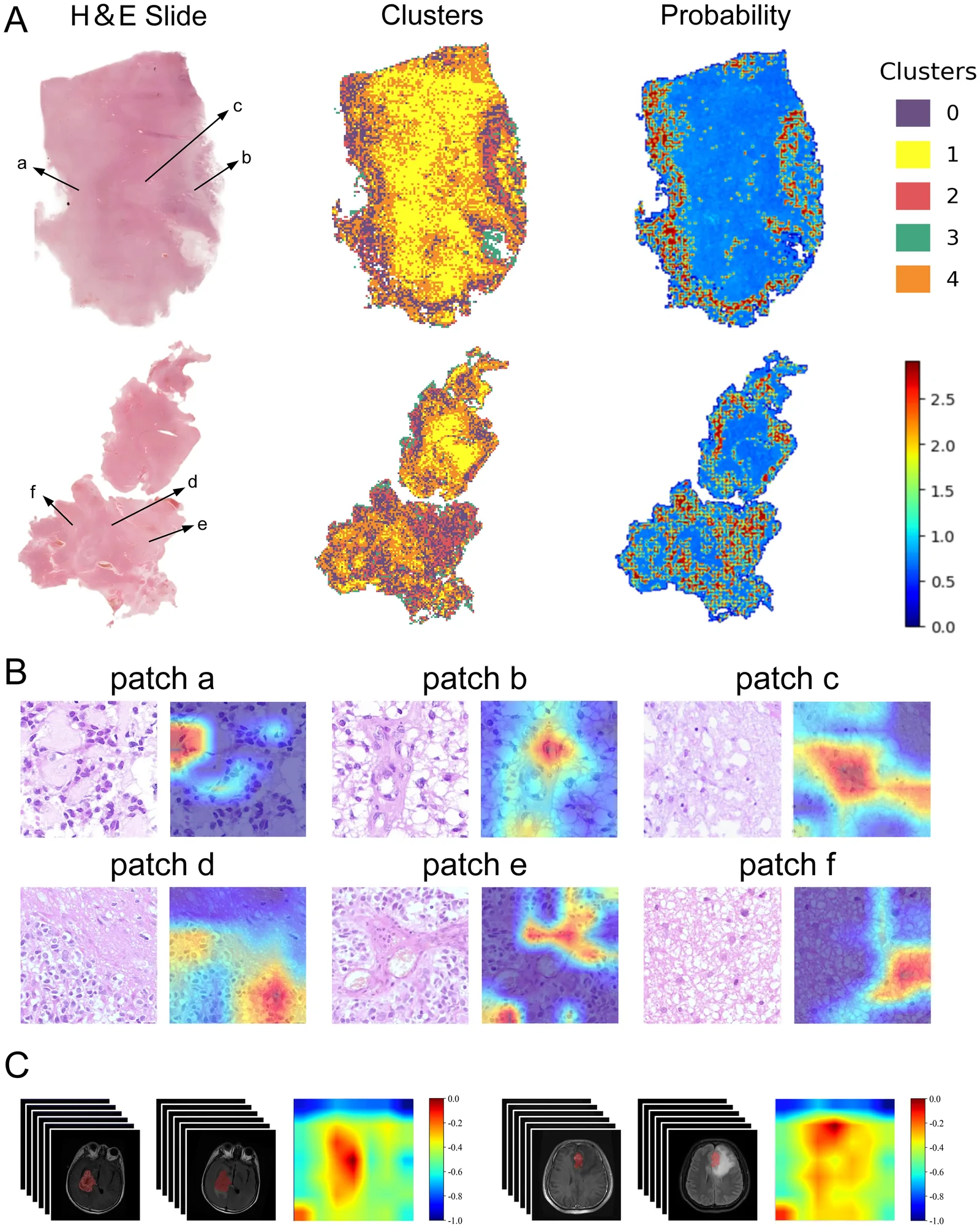
Spatial distribution and representative patches of H&E-stained whole-slide images in the low-benefit group. **(A)** Left column: H&E-stained whole-slide images; second column: patch clusters based on deep learning features; third column: a graph generated based on distances between clusters. **(B)** Representative patches from H&E-stained sections. Patches a and d correspond to cluster 0, patches b and e correspond to cluster 2, and patches c and f correspond to cluster 4, each overlaid with the corresponding Grad-CAM heatmap highlighting regions of interest identified by the deep learning model. **(C)** 2.5D magnetic resonance images and Grad-CAM heatmaps.

**Figure 5:**
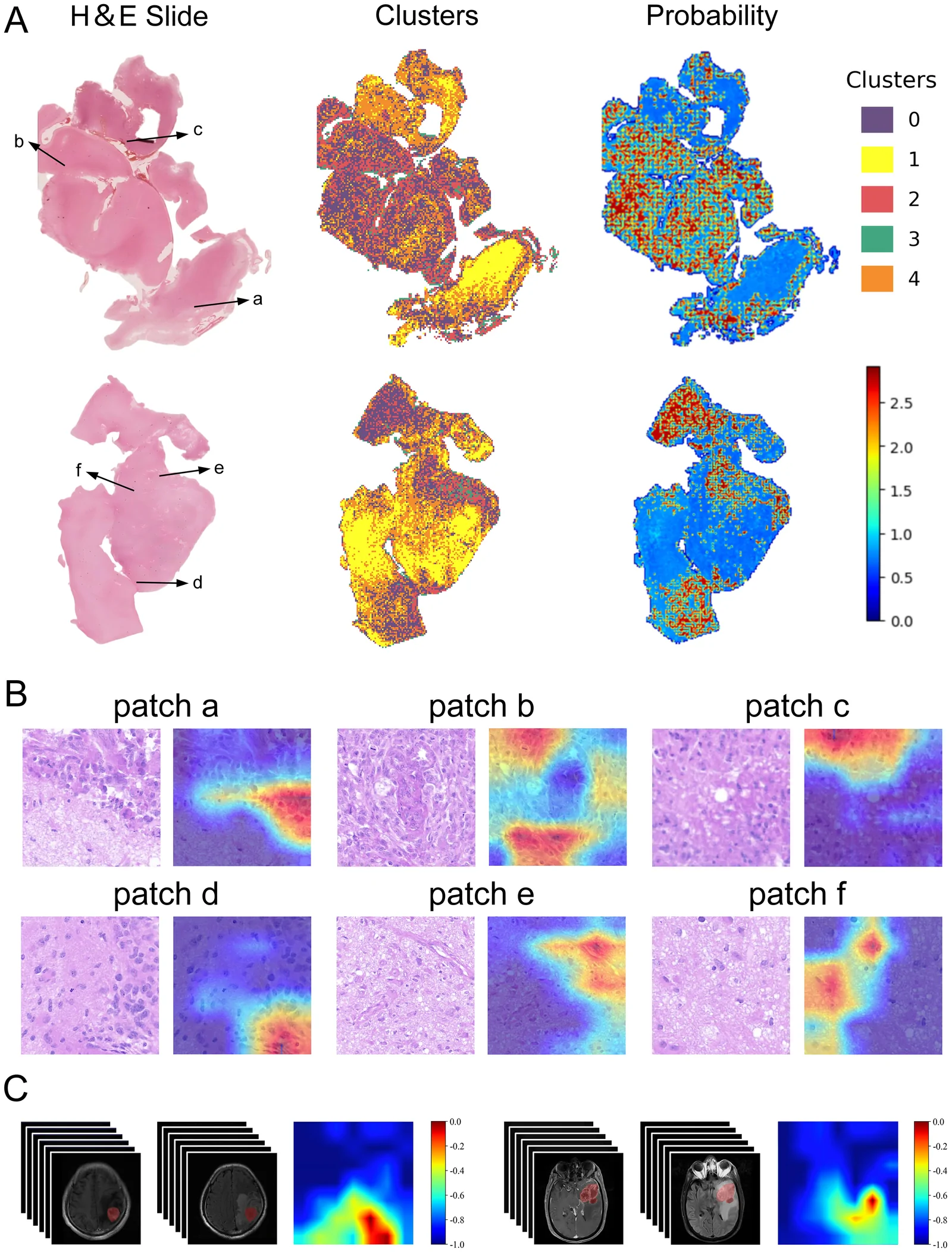
Spatial distribution and representative patches of H&E-stained whole-slide images in the high-benefit group. **(A)** Left column: H&E-stained whole-slide images; second column: patch clusters based on deep learning features; third column: a graph generated based on distances between clusters. **(B)** Representative patches from H&E-stained sections. Patches a and d correspond to cluster 0, patches b and e correspond to cluster 2, and patches c and f correspond to cluster 4, each overlaid with the corresponding Grad-CAM heatmap highlighting regions of interest identified by the deep learning model. **(C)** 2.5D magnetic resonance images and Grad-CAM heatmaps.

### 3.4 Biological validation results

Based on the deep learning prediction model, HGG patients were stratified into high-benefit and low-benefit groups. Comparative transcriptomic analysis identified 942 differentially expressed genes (475 upregulated, 467 downregulated; **Figure 6A**). Significantly upregulated genes included TINAG, CPLX2, and HOXA13, while downregulated genes included INS-IGF2 and CLEC4M (**Supplementary Table S1**). To further elucidate functional differences between groups, GSEA revealed that the high-benefit group was significantly associated with DNA replication, chromatin modification, and RNA expression regulation pathways, including “Assembly of the ORC complex at replication origins” and “Negative regulation of RNA expression by SIRT1” (**Figure 6B**). Clustering analysis further organized these pathways into coordinated regulatory networks (**Figure 6C**). A PPI network constructed from high-frequency non-histone genes selected from core enrichment genes (**Figure 6D**) revealed that DNA damage repair factors, including BRCA1, BRCA2, RAD51, and MRE11, formed highly interconnected hub modules, with topological analysis identifying BRCA1 and RAD51 as central hubs (complete list of high-frequency non-histone genes provided in **Supplementary Table S2**). Functional module analysis resolved this network into three major clusters: Cluster 1 (red, homologous DNA pairing and strand exchange), Cluster 2 (green, DNA replication), and Cluster 3 (blue, nuclear lamina disassembly). These findings provide preliminary characterization of the multi-level molecular pathways associated with model-predicted TMZ treatment benefit, highlighting the potential role of DNA damage repair processes in treatment response.

**Figure 6:**
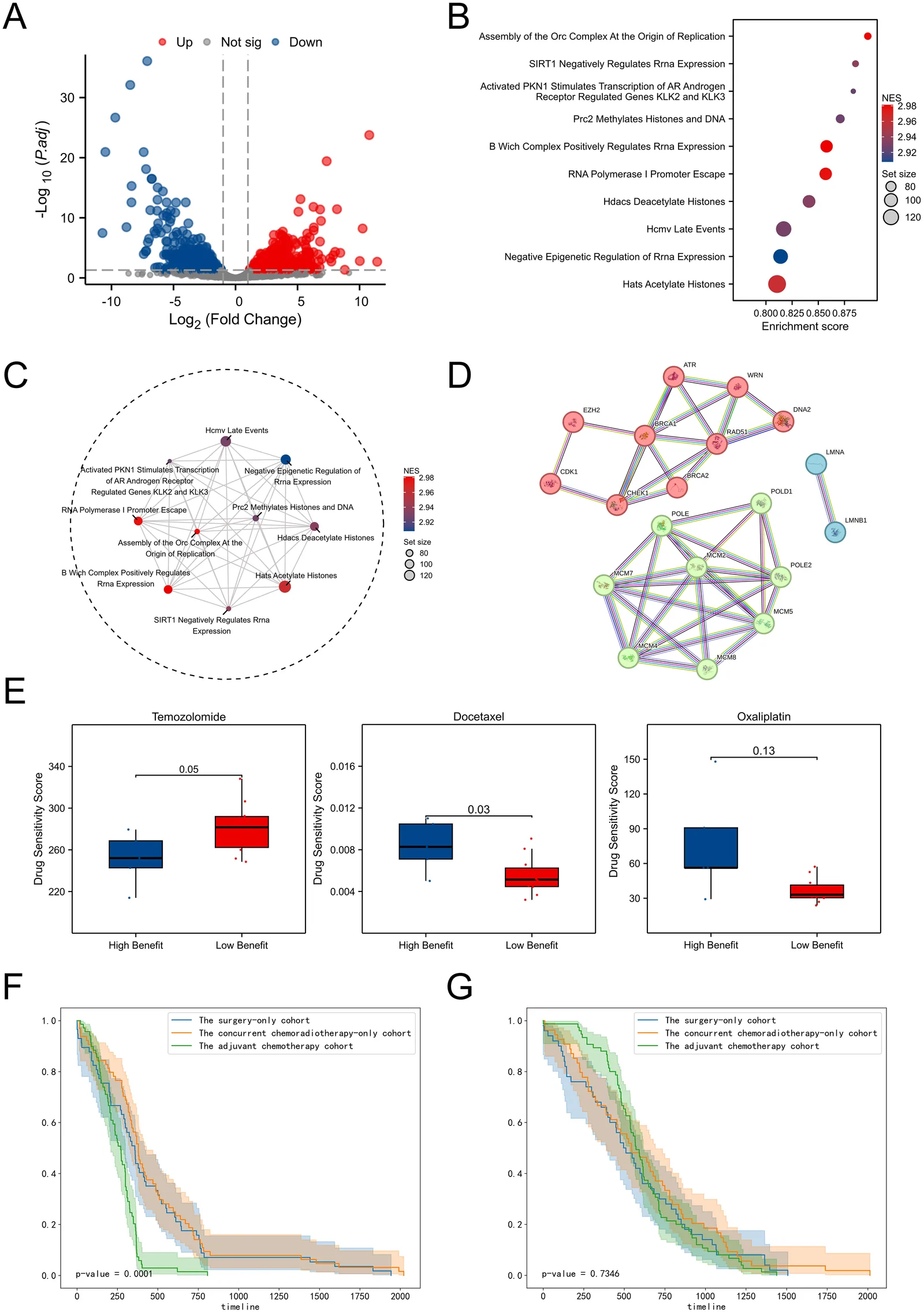
Transcriptomic validation and prognostic evaluation of the model. **(A)** Volcano plot showing differentially expressed genes between the two groups. **(B)** GSEA-enriched pathways, such as DNA replication and RNA expression regulation. **(C)** Clustering network of key pathways. **(D)** Protein-protein interaction network of core non-histone genes and their functional modules. **(E)** In silico drug sensitivity analysis showing differential responses of the two groups to TMZ and other chemotherapeutic agents (docetaxel, oxaliplatin); lower scores indicate higher sensitivity. Kaplan-Meier survival curves comparing treatment groups in predicted low-benefit **(F)** and high-benefit **(G)** patients.

*In silico* drug-sensitivity analysis using oncoPredict (lower scores indicate greater sensitivity) revealed that the high-benefit group showed significantly greater sensitivity to TMZ than the low-benefit group, consistent with the model-predicted survival benefit. In contrast, the low-benefit group exhibited greater sensitivity to docetaxel (P = 0.03) and oxaliplatin (P = 0.13) than the high-benefit group. These observations suggest that alternative chemotherapeutic agents with different mechanisms of action may offer greater therapeutic potential for patients with low predicted benefit from TMZ treatment (**Figure 6E**).

To provide additional immunological context for the model’s biological interpretation, we performed immune deconvolution analyses using three complementary methods on the same transcriptomic cohort (**Figure 7**). ESTIMATE analysis revealed that the high-benefit group exhibited significantly higher ImmuneScore (P = 0.024) and ESTIMATEScore (P = 0.036) compared to the low-benefit group (**Figure 7A**). QuanTIseq deconvolution further showed that the high-benefit group was characterized by significantly higher M1 macrophage infiltration (P = 0.017, FDR-adjusted P = 0.035) and significantly lower regulatory T cell abundance (P = 0.008, FDR-adjusted P = 0.021; **Figure 7B**; **Supplementary Table S6**). Additionally, B cell and dendritic cell fractions were significantly lower in the high-benefit group (both FDR-adjusted P < 0.05; **Supplementary Table S6**), while no significant differences were observed in CD8 T cells, NK cells, or other cell populations (all P > 0.05). MCPcounter analysis additionally revealed a trend toward higher endothelial cell infiltration in the low-benefit group (P = 0.021, FDR-adjusted P = 0.205; **Figure 7C**; **Supplementary Table S7**), consistent with the model’s identification of microvascular proliferation zones as informative pathological regions in low-benefit predictions.

**Figure 7:**
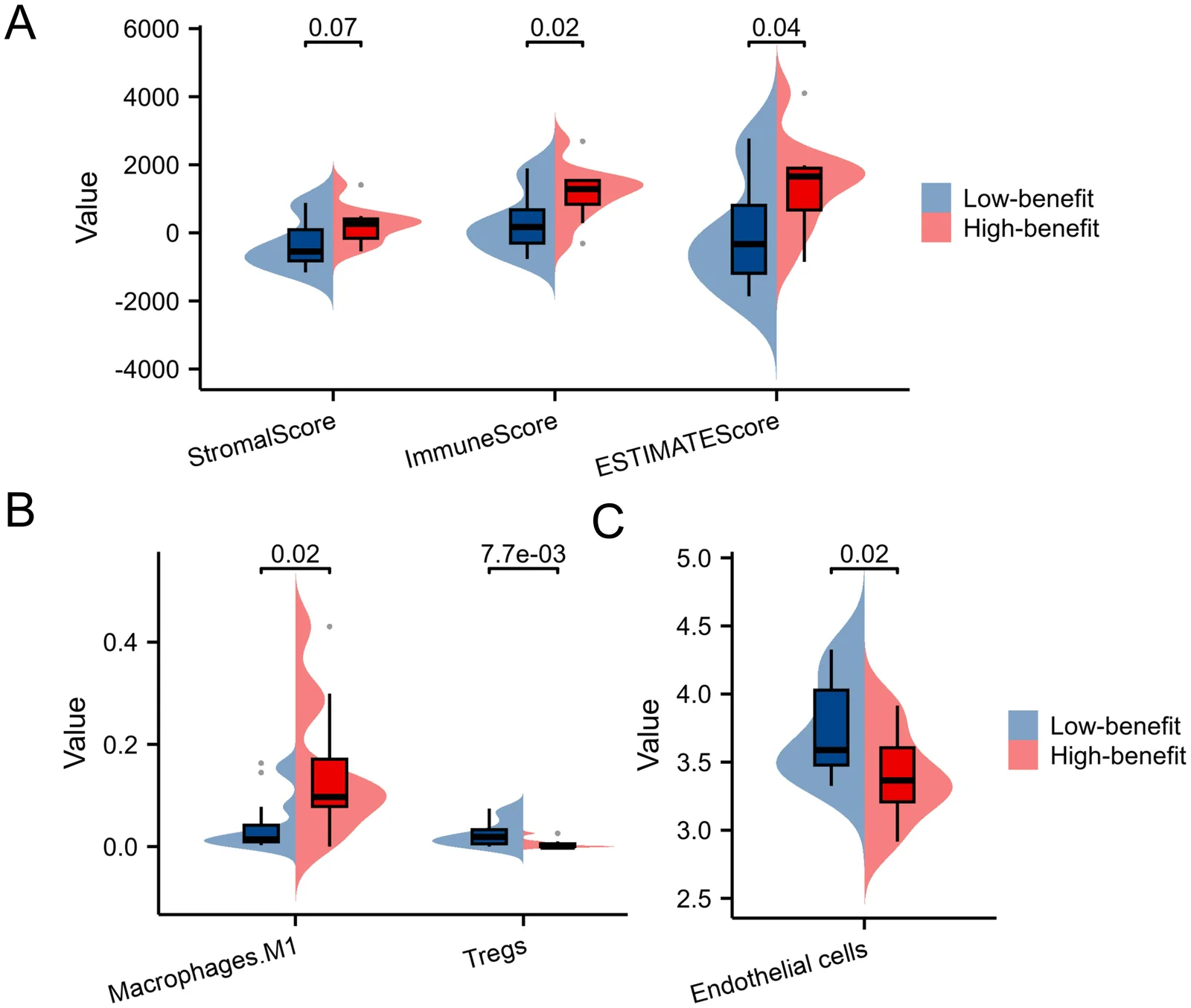
Immune deconvolution analyses comparing model-predicted high-benefit and low-benefit groups in the transcriptomic cohort. **(A)** ESTIMATE analysis showing TumorPurity, ImmuneScore, and ESTIMATEScore comparisons between the two groups. **(B)** QuanTIseq deconvolution results showing the absolute fractions of Macrophages M1 and regulatory T cells (Tregs) between the two groups. **(C)** MCPcounter analysis showing endothelial cell infiltration scores between the two groups.

### 3.5 Prognostic performance evaluation

The multimodal fusion model achieved the highest AUC values across all cohorts: 0.910 in the training cohort, 0.861 in the internal validation cohort, and 0.833 in the external validation cohort. When applied to the concurrent chemoradiotherapy alone and surgery alone cohorts, the Transformer fusion model demonstrated significant risk stratification (**Figures 6F, G**). In the predicted low-benefit subgroup, survival outcomes of patients receiving adjuvant chemotherapy showed a significant separation from both control groups (log-rank test, P < 0.05), whereas no significant differences were observed in the high-benefit subgroup. This survival trajectory analysis suggests that deep learning features based on pathological region selection and 2.5D multi-slice MRI may provide incremental prognostic value for predicting postoperative survival benefit from adjuvant TMZ chemotherapy, supporting further exploration of model-informed postoperative treatment stratification in glioma management. To evaluate the incremental value of the multimodal model over established molecular biomarkers, we conducted AUC comparisons and Cox regression analyses. The model alone achieved an AUC of 0.873, substantially outperforming MGMT (0.615), IDH (0.635), and their combination (0.678). In multivariable Cox regression, the model retained independent prognostic value after adjusting for MGMT and IDH (HR = 0.147, 95% CI: 0.089–0.241, P < 0.001), while MGMT (P = 0.234) and IDH (P = 0.112) both lost significance (**Supplementary Table S4**). Sensitivity analysis further adjusting for WHO grade confirmed these findings (**Supplementary Table S5**).

## 4 Discussion

In this study, we successfully developed and validated a Transformer-based multimodal deep learning model that integrates digital pathological slides with multiparametric MRI to predict postoperative survival benefit from standard adjuvant TMZ chemotherapy in patients with HGGs. Our core findings extend beyond the model’s robust predictive performance demonstrated in independent cohorts; crucially, they reveal interpretable imaging and pathological features closely associated with treatment response and the immune-related tumor microenvironment, elucidate underlying molecular biological mechanisms including DNA repair and antitumor immunity, and identify a specific patient subgroup that may potentially be “harmed” by standard chemotherapy. This provides a novel perspective and a powerful tool for precision decision-making in the treatment of HGGs.

Traditional prognostic models have predominantly relied on single-modality features from radiomics or pathomics, whereas integrating multimodal data has been shown to enhance predictive performance (20–22). This study confirms the significant advantages of deeply integrating multi-source heterogeneous data, including WSIs and MRI. We designed a “pathological region selection” pipeline that excludes noisy regions such as high cellular density areas and focuses analysis on more biologically meaningful tumor microenvironment regions, including the infiltrative tumor margin, microvascular proliferation zones, and necrotic areas, thereby substantially improving model discriminative accuracy. This suggests that for treatment response prediction, the “marginal behavior” and “microenvironmental status” of tumors provide more informative value than mere cellular density within core regions (23, 24). The Transformer architecture, with its powerful capability for feature interaction and global dependency modeling, offers unique advantages for complex multimodal, high-dimensional data fusion tasks, effectively capturing long-range dependencies across spatially distributed patches and outperforming traditional CNN models that rely solely on local feature representations (25, 26). In our study, although the deep learning features achieved clear visual separation among the five pathological clusters in unsupervised visualization, the performance of CNN classifiers was relatively limited after selecting three of these clusters for supervised classification. This performance gap likely reflects the fundamental architectural difference between CNN and Transformer: while CNN extracts features through local receptive fields that may fail to capture contextual relationships across spatially separated regions, the Transformer’s self-attention mechanism enables global interaction among all patches, thereby producing more discriminative feature representations for cluster-level classification. Grad-CAM heatmaps provided imaging and pathological perspectives for interpreting model predictions, helping to characterize the differences between the two treatment response patterns. From the pathological perspective, Grad-CAM activations in low-benefit patients preferentially attended to the tumor-host tissue interface at the infiltrating front, where aggressive histological features were observed. These margin regions represent critical interfaces in high-grade gliomas, where immunosuppressive cells such as tumor-associated macrophages and regulatory T cells frequently accumulate, contributing to an immunosuppressive microenvironment and enabling tumor immune escape (27–29). In contrast, high-benefit patients showed pathological activations associated with regions exhibiting organized tissue architecture and minimal residual disease, consistent with a less aggressive biological phenotype. From the MRI perspective, imaging Grad-CAM heatmaps in low-benefit patients revealed broader and more diffuse activation patterns extending into surrounding parenchyma, reflecting infiltrative growth with compromised blood-brain barrier integrity and aberrant neovascularization — a pattern that has been recognized as a radiographic surrogate for active immune-vascular remodeling in the glioma microenvironment. The spatial correspondence between these imaging activations and the microvascular proliferation zones identified by our pathological region selection strategy further supports this interpretation. Conversely, imaging heatmaps in high-benefit patients showed more circumscribed activation confined to the peritumoral zone, consistent with a confined growth pattern with maintained structural boundaries, which has been associated with relatively intact host immune surveillance in peritumoral regions (30, 31). The distinct spatial patterns of both pathological and imaging Grad-CAM activation thus provide a multimodal foundation for the divergent tumor microenvironment states subsequently characterized by transcriptomic and immune infiltration analyses.

Transcriptomic analysis provided exploratory biological context consistent with the model predictions. The significant enrichment of DNA replication, chromatin modification, and RNA expression regulation pathways in the high-benefit group, particularly the heightened activity of the homologous recombination repair (HRR) network centered on genes such as BRCA1 and RAD51, suggests a potential role of the DNA damage response system in TMZ treatment (32–37). These findings suggest that high-benefit tumors may maintain a more coordinated and intact DNA repair capacity, consistent with their relatively higher genomic stability. In this context, TMZ-induced damage might more readily trigger canonical cell cycle checkpoint activation and apoptotic signaling pathways, leading to tumor cell elimination, rather than promoting survival through error-prone repair mechanisms that accumulate mutations and subsequently drive resistance (38–40). Notably, an intact DNA damage response also facilitates the release of damage-associated molecular patterns and activation of the cGAS-STING pathway, thereby promoting immunogenic cell death and antitumor immune responses. This inference aligns with the computational drug sensitivity analysis, which showed that the high-benefit group exhibited greater predicted sensitivity to TMZ. Conversely, downregulation of HRR-related pathways in the low-benefit group may indicate a greater reliance on error-prone repair mechanisms in the DNA damage response, potentially leading to an increased mutational burden that could influence the tumor immune microenvironment. Drug sensitivity analysis suggested that low-benefit patients might be more sensitive to docetaxel (a microtubule stabilizer) and oxaliplatin (a platinum-based DNA crosslinking agent), providing valuable alternative therapeutic clues for these “TMZ-refractory” patients and emphasizing the necessity of selecting chemotherapeutic agents with distinct mechanisms of action based on molecular subtypes (41–43). However, we acknowledge that these transcriptomic analyses were performed on a limited subset of 43 patients from the external validation cohort. The relationship between TMZ sensitivity and DNA repair biology is highly complex, involving MGMT, mismatch repair status, and replication stress responses, among other mechanisms. The observed pathway enrichment and differential immune profiles do not establish causal mechanisms, and all transcriptomic findings should be interpreted as hypothesis-generating observations that require validation in larger, prospectively characterized cohorts.

The most clinically significant finding of this study is that, within the model-predicted low-benefit subgroup, patients who received complete adjuvant TMZ chemotherapy had shorter survival than those who received concurrent chemoradiotherapy alone or surgery alone. However, we emphasize that this comparison is based on a retrospective, non-randomized design, and patients who completed full adjuvant TMZ may differ from those who did not in multiple unmeasured ways, including performance status, extent of resection, postoperative complications, and clinician-driven treatment selection. Given the ethical impossibility of prospectively randomizing glioma patients to withhold standard chemotherapy, we designed this survival trajectory analysis as an exploratory framework to evaluate whether model predictions carry prognostically meaningful information across different treatment contexts. The surgery-alone and concurrent chemoradiotherapy-alone cohorts were included as contextual reference groups rather than direct causal comparators. The significant survival separation observed in the predicted low-benefit subgroup, but not in the high-benefit subgroup, suggests that the model captures prognostic information consistent with differential treatment benefit, although this cannot be interpreted as causal evidence. For patients with imaging, pathological, and molecular characteristics associated with predicted low benefit, standard adjuvant TMZ chemotherapy may not provide meaningful survival improvement (44, 45), underscoring the importance of exploring alternative approaches such as enrollment in clinical trials of novel agents, intensification of local therapy, or early integration of supportive care. This represents a step toward more individualized postoperative treatment stratification in glioma management.

Although the limited sample size of the transcriptomic cohort precludes definitive conclusions regarding immune microenvironment composition, the immune infiltration analyses provide additional biological support for the model’s predictions. The significantly higher ImmuneScore and ESTIMATEScore in the high-benefit group suggest a more active overall immune microenvironment in tumors predicted to derive greater survival benefit from TMZ. At the cell-type level, the higher M1 macrophage infiltration and lower regulatory T cell abundance in the high-benefit group are consistent with a more pro-inflammatory and less immunosuppressive microenvironment, which may facilitate TMZ-induced immunogenic cell death. These findings align with the pathological and imaging observations described above, where high-benefit patients exhibited localized tumor growth with relatively preserved host immune responses in peritumoral regions. Furthermore, MCPcounter analysis revealed a trend toward higher endothelial cell infiltration in the low-benefit group (P = 0.021), providing independent immunological support for the pathological region selection strategy’s identification of microvascular proliferation zones as informative regions. Notably, the high-benefit group was also characterized by lower B cell and dendritic cell fractions (both FDR-adjusted P < 0.05), reflecting a distinct immune cell distribution landscape in the glioma microenvironment that warrants further investigation. These immune findings, combined with the DNA damage repair pathway enrichment, suggest that the model captures tumors with fundamentally different microenvironment states that may be associated with both treatment sensitivity and immune contexture.

Encouragingly, our observation that radiopathomic features may be associated with the immunological contexture of gliomas finds convergent support from several recent radiogenomic studies. Wang et al. employed an orthotopic glioblastoma mouse model with Treg depletion to demonstrate that MRI radiomic features causally reflect Treg infiltration abundance, with the radiomic score significantly correlated with hypoxia, glycolysis, and IL-2/STAT5 signaling pathways (46). Chen et al. developed an MRI-based radiomic signature (RIB) for predicting M2-like tumor-associated macrophage density, and further validated that patients with higher RIB scores, indicative of “hot” tumors, derived greater survival benefits from dendritic cell vaccine therapy (47). In a multicenter study, Duan et al. identified three immune subtypes of glioma (inflamed, intermediate, and cold) and established a biologically interpretable radiomic signature associated with angiogenesis, hypoxia, and antigen presentation pathways (48). Collectively, these independent lines of evidence from distinct studies support the notion that MRI- and pathology-derived AI features can capture key characteristics of the tumor immune ecosystem. Notably, in our model, the Grad-CAM attention maps preferentially focused on infiltrating margins and peritumoral edema regions, both of which are recognized regions of active immune cell trafficking and microenvironmental remodeling in gliomas — the same biological processes that underpin the radiomic features identified by the aforementioned studies. The spatial concordance between model decision regions and these immune cell-enriched niches provides additional biological plausibility for interpreting model predictions as indirect indicators of the glioma immune microenvironment.

The limitations of this study include its retrospective, multicenter design, which precluded uniform availability of several clinically relevant variables—including 1p/19q codeletion, extent of resection, KPS/ECOG performance status, and detailed treatment parameters—across all cohorts. Although the multimodal model demonstrated robust prognostic performance after adjusting for available molecular markers (MGMT and IDH) and WHO grade, the potential confounding effects of unmeasured variables warrant cautious interpretation and necessitate validation through prospective studies with standardized data collection. The model’s dependence on postoperative pathology also requires further validation in preoperative biopsy cohorts. Furthermore, the immune infiltration analyses presented here are exploratory and based on a limited transcriptomic cohort; future studies with larger cohorts and more comprehensive immune profiling, including single-cell resolution or multiplex spatial immunophenotyping, would help further elucidate the immunological underpinnings of the radiopathomic features captured by our model. Future work will integrate multi-omics data to further elucidate underlying mechanisms and promote clinical translation, assessing its actual impact on clinical decision-making and patient outcomes.

## 5 Conclusion

This study developed an interpretable multimodal deep learning model capable of predicting postoperative survival benefit from adjuvant TMZ chemotherapy in patients with HGGs prior to treatment initiation, with predictions strongly supported by molecular characteristics, including DNA damage repair pathways, which also play a critical role in modulating immunogenic cell death and antitumor immune responses. This not only provides biological interpretability of the model’s predictive performance but, more importantly, enables the identification of a patient subgroup that may not derive meaningful survival benefit from current standard chemotherapy, thereby supporting more informed postoperative treatment decision-making. By linking imaging and pathological features to the tumor immune microenvironment, this research offers a tool with translational potential for precision treatment of gliomas, advocating for future individualized postoperative treatment stratification based on such predictions to guide patients toward alternative approaches with greater likelihood of benefit, including immunotherapy combination strategies.

## Data Availability

The data analyzed in this study is subject to the following licenses/restrictions: The datasets used in this study consist of two categories: Training and internal validation datasets were retrospectively collected from three affiliated hospitals of Shanxi Medical University. These datasets contain protected patient health information and are not publicly available due to institutional review board restrictions and patient privacy protection requirements. Access to these data may be granted upon reasonable request, subject to approval from the Institutional Review Board of Shanxi Medical University and the execution of appropriate data transfer agreements. External validation datasets were obtained from publicly available repositories, including The Cancer Imaging Archive (TCIA) and The Cancer Genome Atlas (TCGA). These datasets are openly accessible under their respective data use policies, which require appropriate citation and compliance with the terms of use specified by the data repositories. Requests to access these datasets should be directed to Corresponding author: Hui Zhang Department of Radiology, First Hospital of Shanxi Medical University 85 Jiefang South Road, Taiyuan 030001, Shanxi Province, China Email: zhanghui_mr@163.com Requests will be considered on a case-by-case basis. Access to the training and internal validation datasets is subject to approval from the Institutional Review Board of Shanxi Medical University and execution of appropriate data transfer agreements to ensure patient privacy protection.
