# Supplementary material for: Pathology-guided multimodal transformer predicts postoperative survival benefit from adjuvant chemotherapy in glioma: from DNA damage repair to immune characterization
Source: Front Immunol. 2026 Oct 16;17:1845251. doi: 10.3389/fimmu.2026.1845251 (PMC13719454; doi:10.3389/fimmu.2026.1845251)
Supplement: Supplementary file 1 [file Supplementaryfile1.docx]

**Supplementary Material**

Pathology-Region Enhanced Transformer for Multimodal Prediction of Chemotherapy Benefit in High-Grade Glioma

**The inclusion criteria were as follows:** (1) patients confirmed by pathology and genetic testing to have grade 3 or 4 gliomas according to the 2021 WHO Classification of Tumors of the Central Nervous System; (2) availability of preoperative MRI (T1-CE and T2-FLAIR) and postoperative pathological slides; (3) complete postoperative follow-up data with documented treatment regimens. **Exclusion criteria comprised:** (1) history of brain tumor biopsy or surgery; (2) absence of T1-CE or T2-FLAIR images, or presence of image quality issues such as severe motion artifacts, metal artifacts, or pathological slide contamination; (3) complications due to other central nervous system diseases including cerebral hemorrhage, cerebral infarction, or Alzheimer's disease; (4) incomplete follow-up information.

**MRI Acquisition Protocol**

All patients underwent preoperative MRI examination on a 3.0-T system (Signa HDxt, GE Healthcare, Milwaukee, WI, USA) with an 8-channel phased-array head coil. The imaging protocol comprised two sequences: (1) contrast-enhanced T1-weighted imaging (T1-CE), acquired with the following parameters: TR/TE = 195/4.76 ms, FOV = 240 mm, slice thickness/inter-slice gap = 5.0/1.5 mm, and acquisition matrix = 256 × 256; and (2) T2-weighted fluid-attenuated inversion recovery (T2-FLAIR), acquired with TR/TE = 8000/95 ms, FOV = 240 mm, slice thickness/inter-slice gap = 5.0/1.5 mm, and acquisition matrix = 256 × 256. Gadolinium-based contrast agent (0.1 mmol/kg body weight) was administered intravenously prior to T1-CE acquisition. Tumor segmentation was performed on axial slices using ITK-SNAP software (version 3.6.0,

http://www.itksnap.org

). The enhancing tumor boundaries were initially delineated on CE-T1WI, and corresponding T2-FLAIR volumes were co-registered for simultaneous evaluation. All contours were independently drawn by a neuroradiologist with over 10 years of clinical experience and subsequently verified by a second senior neuroradiologist with more than 15 years of experience to ensure inter-rater consistency.

**Pathology Slide Acquisition**

Hematoxylin and eosin (H&E)-stained whole-slide images were generated from formalin-fixed, paraffin-embedded (FFPE) tissue blocks obtained during primary tumor resection. All slides were digitized at 20× magnification and saved in mrxs format. Prior to analysis, each WSI underwent rigorous quality inspection to identify and exclude images affected by artifacts of any kind, including tissue folding, air bubbles, staining irregularities, or scanning artifacts. Only WSIs meeting the quality criteria were retained for downstream processing.

**Molecular Testing Methodology**

**MGMT Promoter Methylation Analysis**

MGMT promoter methylation status was determined by pyrosequencing. Genomic DNA was first subjected to bisulfite conversion using the BisulFlash™ DNA Modification Kit (Epigentek, Farmingdale, NY, USA). The converted DNA was subsequently amplified by PCR in a 40 µL reaction volume using the DRR006 Kit (Takara, Shiga, Japan), with thermal cycling as follows: initial denaturation at 94°C for 2 min, followed by 50 cycles of denaturation (94°C, 20 s), annealing (55°C, 20 s), and extension (72°C, 20 s), and a final hold at 72°C for 5 min. Quantitative methylation analysis was carried out on the PyroMark Q96 platform (Qiagen, Hilden, Germany) following the manufacturer's protocol. A total of ten CpG dinucleotides within the MGMT promoter region were interrogated. The methylation level at each CpG site was quantified, and the mean value across all ten sites was calculated for each sample. Samples with a mean methylation level ≥8% were classified as MGMT methylated, while those below this threshold were classified as unmethylated.

**IDH1/IDH2 Mutation Analysis**

IDH1 and IDH2 mutation status was assessed by Sanger sequencing. Genomic DNA was extracted from FFPE tissue sections (5 µm thick) using the TIANamp FFPE DNA Kit (Tiangen Biotech, Beijing, China). Prior to extraction, hematoxylin and eosin–stained slides were reviewed by a pathologist to confirm adequate tumor content (≥70% viable tumor tissue). PCR amplification was performed using GoTaq® Hot Start Green Master Mix (Promega, Madison, WI, USA) with gene-specific primers targeting the IDH1 R132 and IDH2 R172 hotspot regions. Amplification specificity was confirmed by agarose gel electrophoresis, showing a single product of the expected size. Amplicons were then purified enzymatically using Exonuclease I and Antarctic Phosphatase (New England Biolabs, Ipswich, MA, USA). Bidirectional sequencing was conducted with the BigDye Terminator v3.1 Cycle Sequencing Kit on an ABI 3500DX Genetic Analyzer (Applied Biosystems, Foster City, CA, USA). Resulting chromatograms were reviewed using SeqMan software (DNASTAR, Madison, WI, USA), and sequences were aligned against wild-type reference sequences to detect pathogenic mutations at the canonical hotspot codons.

**Figure S1**

Decision curve analysis and calibration curves.(a-c) DCA of Radiomics, Pathomics, and Fusion models in the training (a), test (b), and validation (c) cohorts. (d-f) Calibration curves of the three models in the training (d), test (e), and validation (f) cohorts.

**Figure S2**

Kaplan-Meier Survival Curves by MGMT Methylation (a) and IDH Mutation Status (b) in the Adjuvant Chemotherapy Cohort

**Table Legend**

**Table S1**

List of differentially expressed genes between the high-benefit and low-benefit groups.

**Table S2**

List of high-frequency non-histone genes selected from core enrichment genes for protein-protein interaction network construction.

**Table S3** Distribution of MGMT Methylation and IDH Mutation Status Across Cohorts

| **Variable** | **Training Cohort** | **Internal Validation Cohort** | **The Surgery-only Cohort** | **The concurrent Chemoradiothrapy-only Cohort** | **P value** |
| --- | --- | --- | --- | --- | --- |
| **MGMT** |  |  |  |  | **0.405** |
| **Methylated** | **76 (53.1%)** | **32 (59.3%)** | **62 (57.9%)** | **75 (63.6%)** |  |
| **Unmethylated** | **67 (46.9%)** | **22 (40.7%)** | **45 (42.1%)** | **43 (36.4%)** |  |
| **IDH** |  |  |  |  | **0.964** |
| **Mutant** | **49 (34.3%)** | **17 (31.5%)** | **34 (31.8%)** | **38 (32.2%)** |  |
| **Wildtype** | **94 (65.7%)** | **37 (68.5%)** | **73 (68.2%)** | **80 (67.8%)** |  |

**Table S4** Incremental Value of the Multimodal Model Over Molecular Biomarkers

| **Analysis** | **Predictor** | **AUC** | **HR (95% CI)** | **p value** |
| --- | --- | --- | --- | --- |
| **A. AUC comparison** |  |  |  |  |
|  | **MGMT alone** | **0.615** |  |  |
|  | **IDH alone** | **0.635** |  |  |
|  | **MGMT + IDH** | **0.678** |  |  |
|  | **Model alone** | **0.873** |  |  |
|  | **Model + MGMT** | **0.872** |  |  |
|  | **Model + IDH** | **0.874** |  |  |
|  | **Model + MGMT + IDH** | **0.873** |  |  |
| **B. Cox regression (univariable) (univariable)regression (univariable)** | |  |  |  |
|  | **MGMT (methylated vs unmethylated)** | | **0.649 (0.488–0.863)** | **0.003** |
|  | **IDH (mutant vs wildtype)** | | **0.554 (0.408–0.752)** | **<0.001** |
|  | **Multimodal model (high- vs low-benefit)** | | **0.127 (0.078–0.206)** | **<0.001** |
| **C. Cox regression (multivariable)** | |  |  |  |
|  | **MGMT (methylated vs unmethylated)** | | **0.832 (0.615–1.126)** | **0.234** |
|  | **IDH (mutant vs wildtype)** | | **0.768 (0.554–1.063)** | **0.112** |
|  | **Multimodal model (high- vs low-benefit)** | | **0.147 (0.089–0.241)** | **<0.001** |

**Table S5** Sensitivity Analysis: Multivariable Cox Regression Adjusted for WHO Grade

| **Variable** | **HR (95% CI)** | **p-value** |
| --- | --- | --- |
| **Multimodal model (high- vs low-benefit)** | **0.149 (0.090–0.245)** | **<0.001** |
| **MGMT (methylated vs unmethylated)** | **0.837 (0.619–1.132)** | **0.249** |
| **IDH (mutant vs wildtype)** | **0.792 (0.564–1.111)** | **0.176** |
| **WHO grade (IV vs III)** | **1.117 (0.784–1.592)** | **0.540** |

**Table S6** Immune Cell Composition Estimated by QuanTIseq in High- and Low-benefit Groups

| **Cell Type** | **Low-benefit** | **High-benefit** | **U statistic** | **P value** | **FDR-adjusted P** |
| --- | --- | --- | --- | --- | --- |
| **Tregs** | **0.024 ± 0.025** | **0.004 ± 0.008** | **117** | **0.008** | **0.021** |
| **Macrophages M1** | **0.042 ± 0.056** | **0.142 ± 0.128** | **31** | **0.017** | **0.035** |
| **Dendritic cells** | **0.102** ± 0.046 | **0.041** ± 0.069 | **126** | **0.002** | **0.014** |
| **B cells** | **0.030** ± 0.017 | **0.011** ± 0.009 | **123** | **0.004** | **0.014** |
| **Macrophages M2** | **0.197 ± 0.106** | **0.293 ± 0.132** | **41** | **0.078** | **0.125** |
| **CD8 T cells** | **0.005 ± 0.012** | **0.002 ± 0.005** | **88** | **0.304** | **0.364** |
| **NK cells** | **0.092 ± 0.023** | **0.106 ± 0.064** | **70** | **0.932** | **0.932** |
| **Other** | **0.486 ± 0.133** | **0.401 ± 0.188** | **90** | **0.319** | **0.364** |

**Table S7** Immune Cell Composition Estimated by MCPcounter in High- and Low-benefit Groups

| **Cell Type** | **Low-benefit** | **High-benefit** | **U statistic** | **P value** | **FDR-adjusted P** |
| --- | --- | --- | --- | --- | --- |
| **Endothelial cells** | **3.744 ± 0.362** | **3.396 ± 0.326** | **112** | **0.021** | **0.205** |
| **Neutrophils** | **2.464 ± 0.207** | **2.707 ± 0.516** | **42** | **0.089** | **0.444** |
| **Monocytic lineage** | **3.880 ± 0.610** | **4.250 ± 0.632** | **45** | **0.128** | **0.400** |
| **Fibroblasts** | **5.049 ± 1.174** | **5.405 ± 0.861** | **47** | **0.160** | **0.400** |
| **CD8 T cells** | **1.050 ± 0.254** | **1.260 ± 0.518** | **59** | **0.248** | **0.486** |
| **Myeloid dendritic cells** | **1.696 ± 0.371** | **1.830 ± 0.355** | **53** | **0.291** | **0.486** |
| **T cells** | **1.707 ± 0.248** | **1.821 ± 0.409** | **60** | **0.514** | **0.612** |
| **B lineage** | **1.272 ± 0.178** | **1.254 ± 0.272** | **83** | **0.544** | **0.612** |
| **Cytotoxic lymphocytes** | **1.985 ± 0.366** | **1.945 ± 0.394** | **83** | **0.551** | **0.612** |
| **NK cells** | **1.082 ± 0.088** | **1.116 ± 0.148** | **65** | **0.702** | **0.702** |

**Table S8**	Clinical Characteristics of the External Validation Cohort

Complete data are provided in a separate supplementary CSV file.

**Table S9**	Clinical Characteristics of the Transcriptomic Validation Subset

Complete data are provided in a separate supplementary CSV file.
